# Spontaneous neoplasia in the western clawed frog *Xenopus tropicalis*

**DOI:** 10.17912/micropub.biology.000294

**Published:** 2020-08-31

**Authors:** Makoto Suzuki, Takeshi Igawa, Nanoka Suzuki, Hajime Ogino, Haruki Ochi

**Affiliations:** 1 Amphibian Research Center, Hiroshima University; 2 Institute for Promotion of Medical Science Research, Yamagata University, Faculty of Medicine

**Figure 1.**
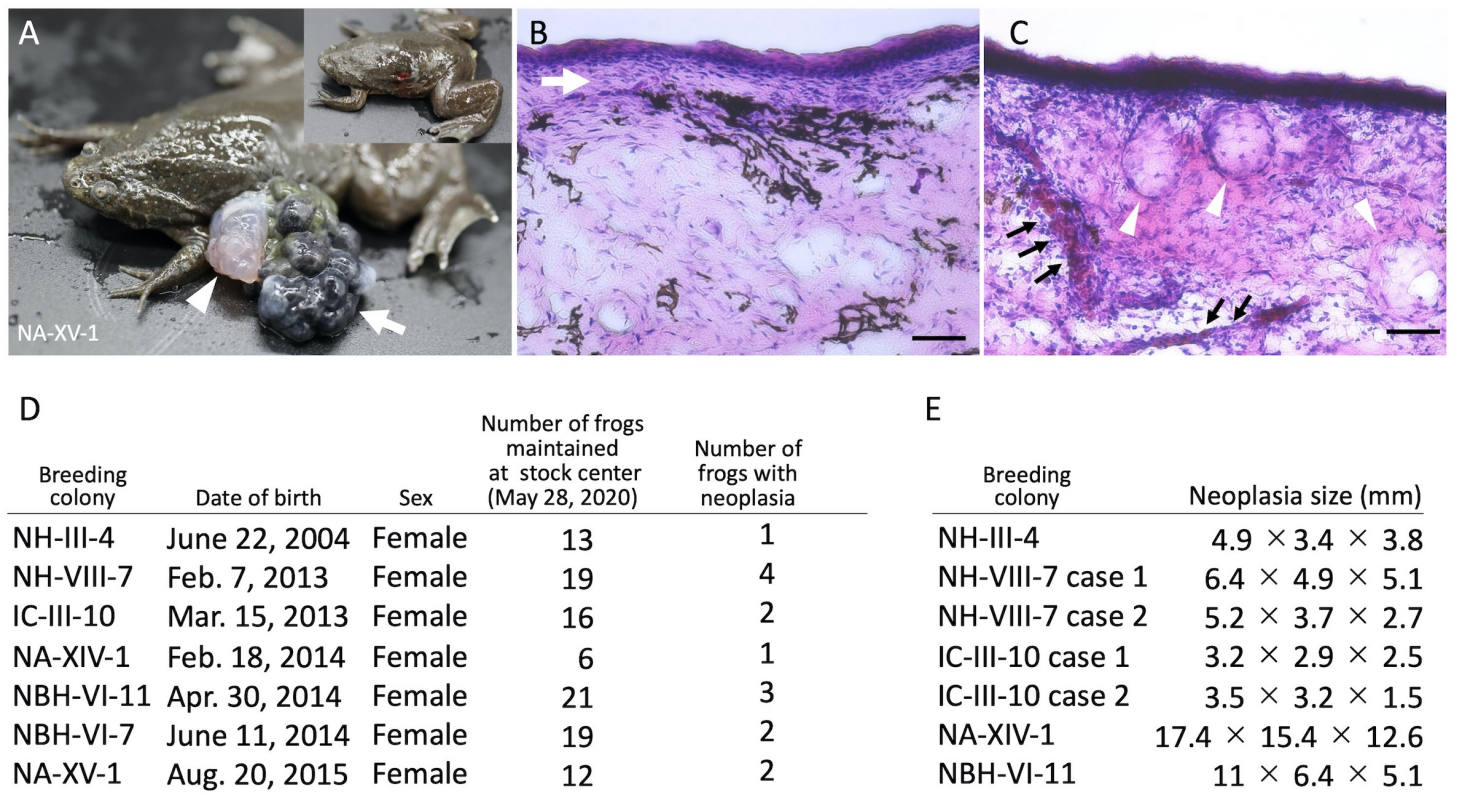
(A–C) Representative images of spontaneous neoplasia in *Xenopus tropicalis*. (A) External morphology of the neoplasia. The arrow indicates the black stone-like nodules, and the arrowhead indicates the white-red nodules. The upper right panel depicts the frog for which the neoplasia was resected. (B) The layered structure in the black stone-like nodules. Hematoxylin–eosin (HE) staining of a histological section of the black stone-like nodule. The white arrow indicates the layered structure. Scale bar indicates 50 μm. (C) The cyst-like structures and blood vessels in the white-red nodule. HE staining of a section of the white-red nodule. The white arrowheads indicate cyst-like structures and the black arrows blood vessels. Scale bar indicates 50 μm. (D) A table showing the number of frogs with neoplasia in each colony. Regarding colony names, the first 2–3 alphabets indicate the strain names, the following Greek numbers indicate the inbred generations, and the Arabic numbers (with case numbers) indicate the colony identifiers. Most of the frogs maintained at the stock center were shipped to users as animal resources, and the remaining frogs as of May 28, 2020, were subjected to this analysis. (E) A table showing the size of neoplasia; the size was determined by measuring diameters at large and small ends, and the height above the skin surface for each neoplasia.

## Description

*Xenopus tropicalis* is an excellent model organism for studies on vertebrate development and regeneration (Horb *et al.*, 2019) and is also useful for the study of tumor formation (Van Nieuwenhuysen *et al.*, 2015; Naert *et al.*, 2016). Spontaneously occurring neoplasia in amphibians have been reported, such as in *X. laevis*, *Rana pipiens*, and *Andria japonicus* (McKinnell *et al.*, 1968; Meyer-Rochow *et al.*, 1991; Kawasumi *et al.*, 2012). In *X. laevis*, one of 4,000 frogs (a 2.5–3-year-old female), which were maintained in artificial outdoor ponds, displayed renal adenocarcinoma without other developmental disorders, indicating that the tumor formation was quite rare in *X. laevis* (Meyer-Rochow *et al.*, 1991). In contrast to *X. laevis*, 3.66 % of R. pipiens in the wild heterogeneous populations had renal adenocarcinoma (McKinnell *et al.*, 1968), which was experimentally induced by herpesvirus (Granoff, 1973). Currently, there are no reports of spontaneous neoplasia in *X. tropicalis*, although recently developed genome editing technologies such as TALEN and CRISPR/Cas9 offer opportunities to induce neoplasia by simple disruption of tumor suppressor genes (Van Nieuwenhuysen *et al.*, 2015; Naert *et al.*, 2016), which would allow modeling of human cancer in this species. The spontaneously occurring neoplasia in *X. tropicalis* could provide excellent opportunities to understand how the genetic background of this species influences the neoplasia phenotypes in combination with disruption experiments of tumor suppressor genes.

The National Bioresource Project (NBRP) of *X. tropicalis* in Japan has successfully developed the four highly inbred wild-type strains, Nigerian A (NA), Nigerian H (NH, previously named Yasuda), Nigerian BH (NBH, previously named Golden), and Ivory Coast (IC) (Igawa *et al.*, 2015). These strains are available for international research communities upon request. We found spontaneous neoplasia formation in the frog stocks at the NBRP (Figure 1). Analysis of frogs from seven colonies with different genetic backgrounds and chronological ages (16, 7, 6, or 5 years) identified neoplasias formed in various parts of the body, including the dorsal side, ventral side, dorsal head, dorsally on limbs, and eye (Figure 1D). Almost all frogs had a single site of neoplasia; just one frog from the NBH-VI-11 colony had multiple sites of neoplasia. The neoplasias of *X. tropicalis* could be divided into two types: black stone-like nodules (Figure 1A, white arrow) and white-red nodules (Figure 1A, white arrowhead). Most of the frogs were harboring only the black stone-like nodules, although a few frogs were harboring both types of nodules simultaneously (Figure 1A). Previous studies have reported that the histological analysis of amphibian and reptile neoplasias revealed the renal mass in *A. japonicus* exhibiting trabecular pattern and the epidermal mass in the *Furcifer pardalis* consisting of concentric keratin material (Kawasumi *et al.*, 2012; Meyer *et al.*, 2019). Histological analysis of *X. tropicali*s neoplasia using hematoxylin–eosin (HE) staining showed that melanocytes were enriched in the black stone-like nodules and that a layered structure was present (Figure 1B, white arrow). In contrast, cyst-like structures and blood vessels were present in the white-red nodules (Figure 1C, white arrowheads and black arrows, respectively). We then measured the neoplasia size (Figure 1E). Two frogs from the NA-XIV-1 colony had a large neoplasia, over 17 mm at the large end, over 15 mm at the small end, and over 12 mm in height (Figure 1E). The average length of the neoplasia was 7.3 mm at the large end, 4.1 mm at the small ends, and 3.5 mm in height. Therefore, it will be of interest if the frequency and size of neoplasia vary among the inbred strains on disruption of tumor suppressor genes via genome editing.

## Methods

Neoplasias were fixed with 4% paraformaldehyde in phosphate-buffered saline (PBS) overnight at 4°C and were successively washed with PBS containing 0.1 % Tween 20. Fixed neoplasias were frozen in Optimum Cutting Temperature Compound, and cryosectioned with a thickness of 10 μm. Sections were stained with HE by a standard method. Each slide was photographed under a Zeiss Axio Vert. A microscope using an AxioCam MRc digital camera.

## Reagents

*X. tropicalis* strains used in this experiment were maintained at the Amphibian Research Center, Hiroshima University. All treatment procedures for *X. tropicalis* were approved by Yamagata University Animal Research Committee (#310300). For HE staining, we used the following reagents: Mayer’s hematoxylin solution and 1% eosin Y solution (FUJIFILM Wako Pure Chemical Corporation, Osaka, Japan), Tissue-Tek optimum cutting temperature compound (Sakura Finetek, Tokyo, Japan).

## References

[R1] Granoff A (1973). Herpesvirus and the Lucké tumor.. Cancer Res.

[R2] Horb M, Wlizla M, Abu-Daya A, McNamara S, Gajdasik D, Igawa T, Suzuki A, Ogino H, Noble A, Robert J, James-Zorn C, Guille M, Centre de Ressource Biologique Xenope team in France. (2019). *Xenopus* Resources: Transgenic, Inbred and Mutant Animals, Training Opportunities, and Web-Based Support.. Front Physiol.

[R3] Igawa T, Watanabe A, Suzuki A, Kashiwagi A, Kashiwagi K, Noble A, Guille M, Simpson DE, Horb ME, Fujii T, Sumida M (2015). Inbreeding Ratio and Genetic Relationships among Strains of the Western Clawed Frog, Xenopus tropicalis.. PLoS One.

[R4] Kawasumi T, Kudo T, Une Y (2011). Spontaneous nephroblastoma in a Japanese giant salamander (Andrias japonicus).. J Vet Med Sci.

[R5] McKinnell RG, McKinnell BK (1968). Seasonal fluctuation of frog renal adenocarcinoma. Prevalence in natural populations.. Cancer Res.

[R6] Meyer-Rochow VB, Asashima M, Moro SD (1991). Nephroblastoma in the clawed frog Xenopus laevis.. J Exp Anim Sci.

[R7] Naert T, Colpaert R, Van Nieuwenhuysen T, Dimitrakopoulou D, Leoen J, Haustraete J, Boel A, Steyaert W, Lepez T, Deforce D, Willaert A, Creytens D, Vleminckx K (2016). CRISPR/Cas9 mediated knockout of rb1 and rbl1 leads to rapid and penetrant retinoblastoma development in Xenopus tropicalis.. Sci Rep.

[R8] Van Nieuwenhuysen T, Naert T, Tran HT, Van Imschoot G, Geurs S, Sanders E, Creytens D, Van Roy F, Vleminckx K (2015). TALEN-mediated apc mutation in Xenopus tropicalis phenocopies familial adenomatous polyposis.. Oncoscience.

[R9] Meyer, J.; Kolodziejek, J.; Häbich, A. C.; Dinhopl, N.; Richter, B., 2019: Multicentric Squamous Cell Tumors in Panther Chameleons <i>(Furcifer pardalis</i>). <i>Journal of Exotic Pet Medicine</i>., <b>29</b>, 166–172.

